# Human–wildlife conflict in the roof of the world: Understanding multidimensional perspectives through a systematic review

**DOI:** 10.1002/ece3.7980

**Published:** 2021-08-02

**Authors:** Prashanti Sharma, Nakul Chettri, Kesang Wangchuk

**Affiliations:** ^1^ International Centre for Integrated Mountain Development Kathmandu Nepal

**Keywords:** cooperation, human–wildlife conflict, knowledge gaps, research trends, spatial and temporal coverage

## Abstract

Human–wildlife conflicts have intensified by many folds and at different levels in recent years. The same is true in the case of the Hindu Kush Himalaya (HKH), the roof of the world, and a region known for its wealth in biodiversity. We present a systematic literature review (SLR) using the search, appraisal, synthesis, and analysis (SALSA) framework; and for spatial and network analysis, we employed the VOSviewer software. The review—covering 240 peer—articles within a span of 27 years (from 1982 to 2019)—revealed that in the last decade, there was a 57% increase in publications but with a disproportionate geographical and thematic focus. About 82% of the research concentrated on protected areas and large carnivores and mega herbivores played a big role in such conflicts. About 53% of the studies were based on questionnaires, and the main driver reported was habitat disturbance of animals due to land‐cover change, urbanization, and increase in human population. On the management front, the studies reported the use of traditional protection techniques like guarding and fencing. Our analysis of 681 keywords revealed a prominent focus on ‘human‐wildlife conflict,’ ‘Nepal,’ ‘Bhutan,’ ‘Snow Leopard,’ and ‘Leopard’ indicating the issue linked with these species and countries. The involvement of 640 authors from 36 countries indicates increasing interest, and Nepal and India are playing key roles in the region. As for the spatial analysis that was conducted, while it showed regional variations, there were conspicuous limitations in terms of having a transboundary focus. Thus, particular attention ought to be paid to building transboundary partnerships and improving management interventions; there is also a pressing need to understand the patterns of human–wildlife convergence, especially involving meso‐mammals.

## Graphical abstract

We present a systematic review on human–wildlife conflict from the roof of the world. The review presents results from 240 peer‐reviewed articles with an increasing trend of publications. About 82% of the research reported cases from protected area indicating conservation challenges. Scholars highlight habitat disturbance through land‐cover change, urbanization, and human population increase as major drivers. More focus on pattern of interactions at transboundary level and effective mitigation measures are recommended.

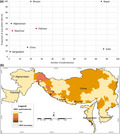

## 1 INTRODUCTION

The interactions between wildlife and human beings have often resulted in agonistic behavior and conflicts (König et al., 2020; Nyhus, 2016). While instances of human–wildlife conflict (HWC) date back to prehistoric times (Berger & McGraw, 2007; Gordon, 2009), its severity and complexity have increased in the current era (Madden, 2004; Sharma et al., 2020). The animals are known to launch lethal attacks on humans, damage property, raid crops, and kill livestock; on the contrary, humans indulge in retaliatory killings, hunting, and poaching—and these could even involve endangered or keystone wildlife species, thereby posing a threat to biodiversity and imposing legal issues on humans (Peterson et al., 2010; White & Ward, 2011). HWC, thus, has led to economic and psychological disruption, as well as to the spread of zoonotic diseases; it also raises the spectre of extinction as far as certain wildlife species are concerned (Barua et al., 2013; Nyhus, 2016; Thirgood et al., 2005).

The reasons behind HWC are multiple: In the case of the wild animals, it is their habitat loss and its degradation owing to urbanization, intensification of agriculture, and growth in human population (Nyhus, 2016)—increased human dominance in natural landscapes intensifies competition for space and resources, especially for large carnivores like the Royal Bengal tiger (*Panthera tigris tigris*) and the common leopard (*Panthera pardus*)—that have led to their antagonistic behavior (DeFries et al., 2010; Zimmerman et al., 2010); while in the case of humans, it is primarily the raiding of their crops by the animals—due to food shortage (Hill, 2018) and habitat fragmentation (Choudhury, 2004) that has led to their confrontational posture (Acharya et al., 2017). It is then obvious that the mitigation of this conflict is central to human safety and the health of the ecosystem; but this requires a profound understanding of interrelated social–ecological relations (Carter & Linnell, 2016; Treves et al., 2006).

Globally, research on HWC and the coexistence of humans and wildlife has exponentially grown over the last decade in the form of peer‐reviewed articles and reports (Holland et al., 2018; König et al., 2020; Nyhus, 2016). According to a recent study, over the last decade, 87% of the publications on HWC concentrated on the Asian countries of India, Nepal, and Indonesia (Torres et al., 2018). This region accounts for the richest collection of earth's biological diversity, but this is being continuously threatened by the expansion of agriculture and overexploitation of wildlife (Monastersky, 2014; Sodhi & Brook, 2006). The Hindu Kush Himalaya (HKH), stretching across eight countries (Afghanistan, Bangladesh, Bhutan, China, India, Myanmar, Nepal, and Pakistan), is the highest, youngest, and one of the richest in terms of species, genetic, and ecosystem diversity among the global mountain biomes (Xu et al., 2019). Indeed, this roof of the world is home to four of the 36 global biodiversity hotspots—the Himalaya, Indo‐Burma, the mountains of Southwest China, and the mountains of Central Asia (Mittermeier et al., 2011). However, in recent years, the HKH has been experiencing rapid demographic and economic growth leading to overexploitation of natural resources; this has resulted in significant Land Use/Land Cover (LULC) changes and in forest loss (Xu et al., 2019). The loss of the region's core forest areas meant a reduction in the dispersal ability of wildlife in their home ranges, thereby forcing them to move into human territory (Acharya et al., 2017). In the HKH, this problem is rather prominent in India, Nepal, and Bhutan in the form of crop‐raiding monkeys and human‐eating tigers (Sharma et al., 2020). In Nepal, for example, between the years 2010 and 2014, on average, as many as 115 people were attacked annually by large mammals such as the Asian elephant (*Elephas maximus*), the Royal Bengal tiger, the Asian black bear (*Ursus thibetanus*), and the common leopard (Acharya et al., 2016). The shrinking of animal habitat also poses threat to the animal's own life, as in the case of India's West Bengal state, where, from 2004 to 2015, 62 elephant fatalities were reported; these elephants were hit by trains that were running on tracks through forest corridors (Roy & Sukumar, 2016).

Several authors employed different scientific approaches to identify the sources and causes of HWC and the means to mitigate it (Acharya et al., 2017; Bashir et al., 2018; Sarker & Røskaft, 2010). The literature that has been published covers various dimensions of HWC, such as those related to crop and property damage, compensation and insurance schemes, people–park relations, and the threat to biodiversity (Aryal et al., 2014; Carter et al., 2014; Huang et al., 2018; Limbu & Karki, 2003).

While it is a fact that diligent efforts are being made by government bodies, research organizations, NGOs, and local communities to tackle HWC, most of their efforts are limited in scope as they are country‐ and location‐specific. The transboundary nature of HWC is an aspect that has been less recognized in the HKH. Moreover, as observed by Wester et al. (2019), countries in the region suffer from inadequate and scattered knowledge generation, which is a major hindrance to understanding the underlying drivers and effects of HWC; this also limits efforts at collaborative natural resource governance (Davies & White, 2012). So, to arrive at a profound comprehension of the “transboundary‐ness” of the HKH, a systematic review and analysis of the existing information became inevitable. Such a review and analysis were bound to provide a holistic insight into the region's knowledge base, its information gaps, and its priority areas for future interventions (Kandel et al., 2016). Besides, the findings of this review and analysis could foster regional learning and cooperation. Taking all these factors into consideration, a systematic review of the literature on HWC in the HKH was thus conducted with two main objectives guiding it. The first objective was to characterize and analyze the scientific literature on HWC according to its spatial and temporal distribution, the scale and theme of the research, its methodological tools and approaches, taxonomy, the drivers of change, and management actions. The second objective was to analyze the collaborative network of research through the study of keywords, coauthorship links, and partnerships among countries to better understand research trends, priorities, alliances, and knowledge gaps.

## 2 METHODS

**FIGURE 1:**
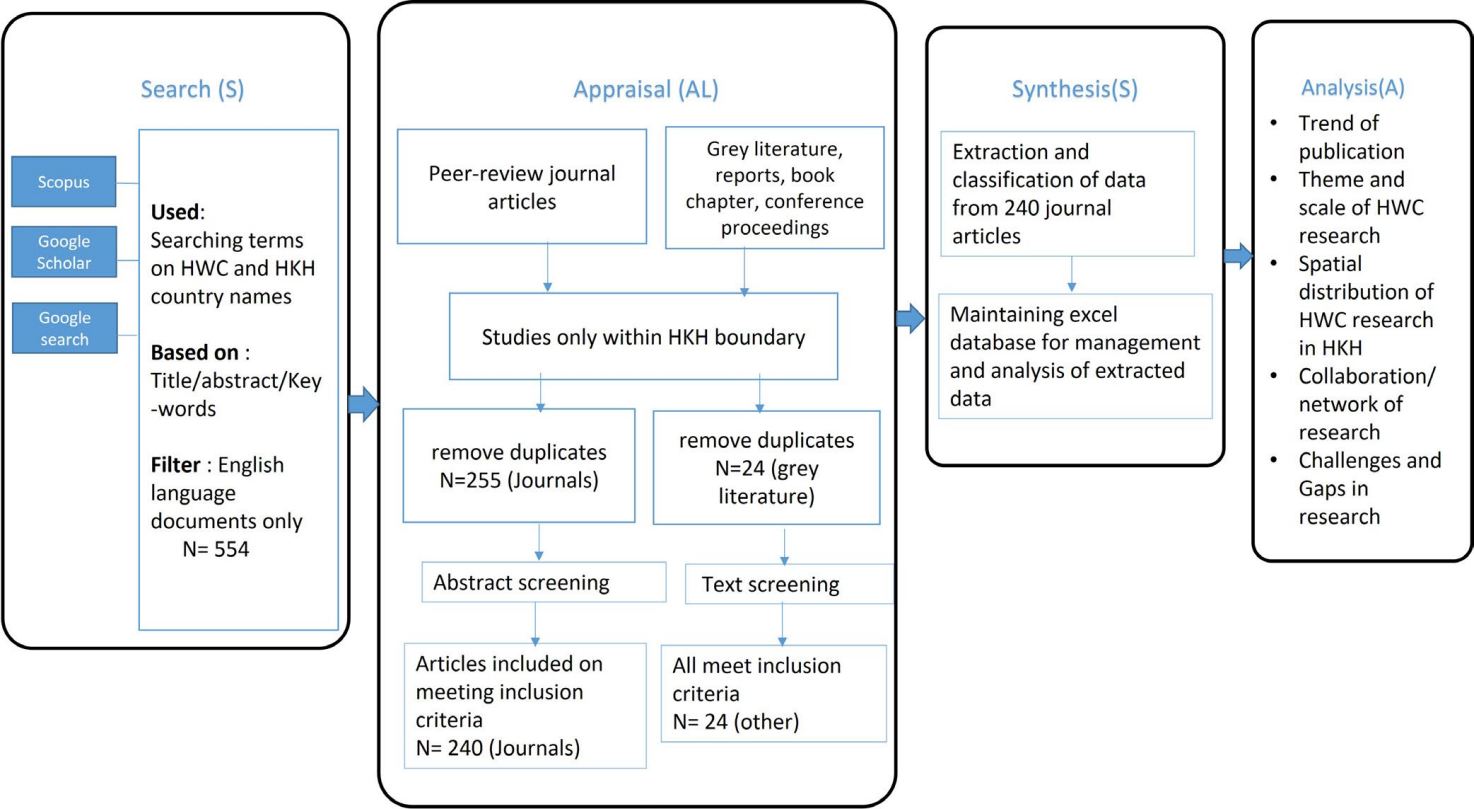
Flow diagram for systematic literature review using Search, Appraisal, Synthesis and Analysis (SALSA) framework

We followed the systematic literature review (SLR) approach of qualitative content analysis, as it is systematic, explicit, and reproducible for identifying, evaluating, and synthesizing the existing body of scientific information (Fink, 2019). The review was conducted using the framework of Grant and Booth (2009), which involved four sequential steps (Figure 1): search, appraisal, synthesis, and analysis (SALSA). The steps of the SALSA framework are explained in Table 1. This method is accurate, systematic, exhaustive, and reproducible (Mengist et al., 2020; Vicente‐Sáez & Martínez‐Fuentes, 2018).

**TABLE 1:** The SALSA framework (Grant & Booth, 2009) used in the systematic review of scientific literature

Step	Outcome	Methods
Search	Search strategy Literature selection criteria	Exhaustive string search
Appraisal	Quality assessment and selection	Inclusion and exclusion of studies based on criteria
Synthesis	Extraction and categorization of data	Graphical and tabular representations
Analysis	Data analysis	Quantitative categories and narrative analysis

### 2.1 Search

In this step, the relevant sources of information were identified from various databases using appropriate search strings. The search databases were Scopus (Elsevier), Google Scholar, and the Google search engine. We opted for Scopus since it is the largest database of peer‐reviewed literature and has more indexed journals (Mongeon & Paul‐Hus, 2016), while Google Scholar and the Google search engine were used to collect all the relevant peer‐reviewed articles and gray literature (reports, conference proceedings, perspectives, keynotes, and book chapters) which were not indexed in Scopus. The term “human–wildlife” conflict in this paper refers to both direct interactions of humans with wildlife through encounters and livestock depredation, and indirect relationships expressed via people's attitudes/perceptions and their sense of well‐being (Lozano et al., 2019). Therefore, we used various combinations of search strings for an exhaustive and comprehensive literature search covering the broad dimensions of HWC. For example, for Nepal, we used the advanced search filter in Scopus with keyword strings: “Human–wildlife conflict” and “Nepal”. A similar search was carried out for all the other seven countries of the HKH (Afghanistan, Bangladesh, Bhutan, China, India, Myanmar, and Pakistan) which formed our study area. Besides, we searched for names of administrative divisions within countries—for instance, “Nepal” and “Chitwan” districts. The search string was also extended to include conflicts involving specific species or families of wildlife for each of the countries of the HKH and its administrative divisions, for instance: “human–carnivore conflict”, “human– monkey conflict”, “human‐elephant conflict”, and “human–rhino conflict”. Moreover, to include the dimension of livestock depredation and crop damage by the animals, keywords such as “wildlife crop raid”, “livestock depredation”, and “animal attack” were used against each of the country names; this also narrowed down the volume of literature to the region of interest. The systematic search for these strings was based on the literature's title, abstract, and keywords and was carried out until December 2019 with no lower‐year limit. Our search was restricted to English‐language articles for this study. For the literature search on Google Scholar and the Google search engine, we employed a similar strategy, mostly aimed at retrieving gray and unindexed literature.

### 2.2 Appraisal

The appraisal phase was about selecting the literature through a screening process. A total of 554 literature data, including peer‐reviewed journal articles and gray literature, were collected from various database sources. The initial step involved separating all the gray literature from the published peer‐reviewed journal articles. We then selected the studies that were exclusively conducted within the HKH boundary (note that countries like India and Bangladesh have a large proportion of such studies outside the HKH). On acquiring the literature data from within our study region, we removed all duplications, which resulted in a total of 255 journal articles and 24 pieces of gray literature. These were then selected for abstract screening.

A total of 240 out of the 255 journal articles fulfilled the eligibility criteria for the final database. The literature removed after the abstract screening was on the basis that these research works did not directly adhere to HWC. All the 24 pieces of gray literature qualified to be included in the final database.

### 2.3 Synthesis

The qualitative approach to synthesize the derived knowledge helps to explore, interpret, and present new perspectives on the acquired data (Vicente‐Sáez & Martínez‐Fuentes, 2018). Hence, in this step, we extracted the relevant data relating to HWC from the 240 journal articles. These data were then maintained and managed in MS Excel for processing. Table 2 shows the categorization of the extracted data into various classes and variables of interest; this was done to meet the SLR objectives. These data were further used for analysis through tabular and graphical representations.

**TABLE 2:** Categorization of information from the selected articles according to various criteria

Criteria	Categories considered	References
Temporal trend of research	Earliest year of publication until December 2019	Nyhus (2016), Kandel et al. (2020)
Spatial pattern of research: Study site (scale and regime)	Research study sites in terms of – Scale: Local level—less than 1,000 km^2^; country level—countrywide/various divisions within country; transboundary level—between member countries Regime: Within and along the periphery of PAs and within corridors and unprotected areas	Mengist et al. (2020)
Types of conflict	Crop and livestock damage; threat to biodiversity and human safety; human–human; and property damage	Peterson et al. (2010), Lozano et al. (2019)
Methods of data collection	Interviews and focus group discussions; biological sign surveys; direct observations; camera trapping; GIS‐based satellite images; GPS radio‐collaring; and secondary sources	Rashid et al. (2020), Lozano et al. (2019)
Approaches in data analysis	Statistical analysis; spatial mapping; statistical modeling; and molecular tracking	N/A
Wildlife's taxonomy	Large carnivores; mega herbivores; herbivores; omnivores; meso‐mammals; medium carnivores; and small carnivores	Peterson et al. (2010)
Drivers of change	Human disturbance; forage/prey availability; proximity to forest; weak policy enforcement; cultural links; and climate change	Lozano et al. (2019)
Management actions	Agricultural and livestock safeguarding strategy; community intervention; plans and policies; and direct intervention	Holland et al. (2018)
Perception, attitude, and gender	Inclusion of perception, attitudes, and gender (Yes/No)	NA

### 2.4 Analysis

This phase involved evaluating the synthesized data to gain meaningful information and answers to the research questions. The categories were quantified and analyzed to explain the results (Table 2). This further paved way for discussions and indicated knowledge gaps in HWC in the region. The study also applied VOSviewer (https://www.vosviewer.com), a desktop‐based, open‐source software, for constructing and visualizing bibliometric networks (Van Eck & Waltman, 2010). The VOSviewer made use of comma‐separated values (CSV) format of the database comprising 240 selected articles. We then investigated the HWC research collaboration network among the various countries and authors in the HKH and also visualized the frequency of keywords to analyze the most researched areas related to HWC.

A map created in the VOSviewer consisted of one type of item (country names, keywords, or author names) connected by lines or links. Each link has a strength, which is represented by a positive numerical value. The strength of a link may, for example, indicate the number of publications two researchers have coauthored (in the case of coauthorship links), the number of publications where the same keywords have occurred together (in the case of keyword co‐occurrence), and the number of publications in which two countries have collaborated (in the case of country coauthorship). A closely linked set of items forms clusters that are linked to other clusters which then constitute a network. The size of each item in a network is weighted by the number of documents, citations, or link strength between two items. The color of an item is determined by the cluster to which the item belongs (Van Eck et al., 2013). We used the number of documents as a weight for calculating the size of the items in mapping keywords, authors, and countries’ networks for HWC in the HKH.

## 3 RESULTS

### 3.1 Temporal and spatial pattern

**FIGURE 2:**
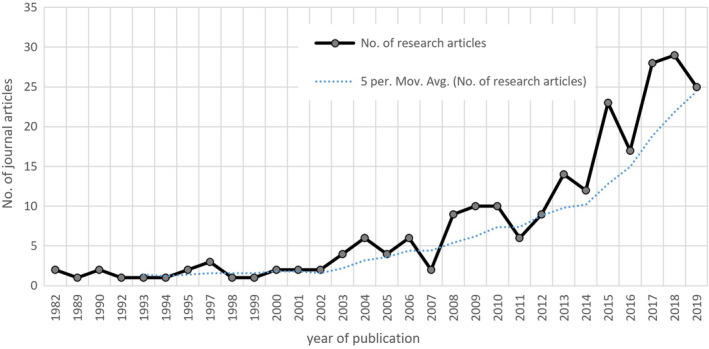
Number of published peer‐reviewed research articles on human–wildlife conflict from 1982 to 2019 in Hindu Kush Himalaya. Trendline represents five‐year moving average indicating increasing volume of publication

In the HKH, the research on the conflict between humans and wildlife saw steady growth during the review period of 1982–2019. Over those 37 years, the number of articles rose from a meager two in 1982 to a healthier 25 in 2019 (Figure 2). The largest number of 30 research articles were published in 2018. The progress of research shown by the overall trend could be grouped into three specific phases: Phase I (1982–2002), where notably, only three research papers were published in 1997—this phase constituted 9% of the total publications under review; Phase II (2003–2008) saw a slight increase, by 4%, in the number of research papers compared with the previous phase—with two publications in 2007 and nine in 2008, thereby suggesting an erratic phase; and Phase III (2009–2019) witnessed an exponential growth in HWC research publications in the HKH with an average increase of 1.5 articles per year—this period of 10 years accounted for 78% of the publications and an increase by 57% compared with the previous two phases.

**FIGURE 3:**
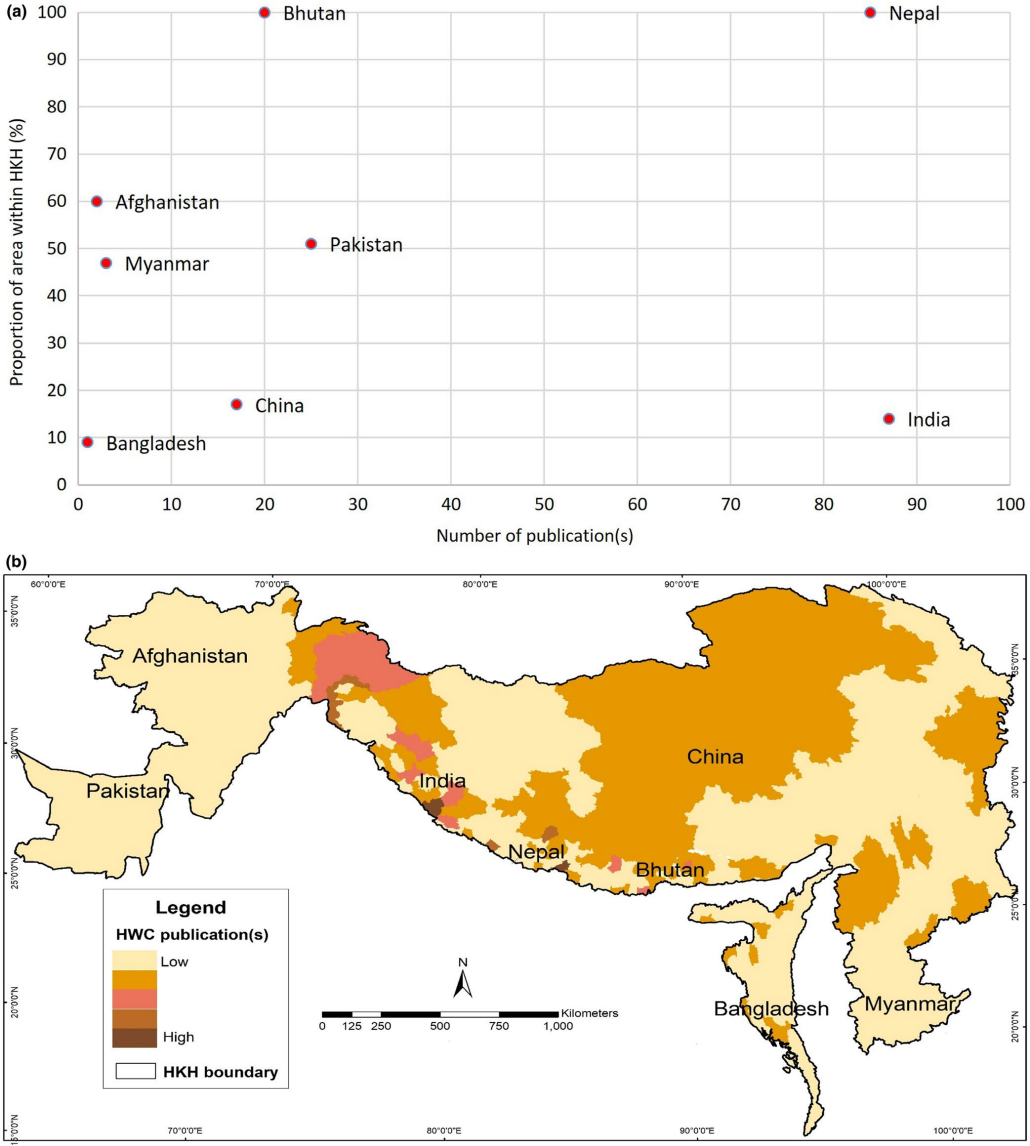
(a) Number of published peer‐reviewed research articles produced by each country verses the percentage of country's area under Hindu Kush Himalaya and (b) spatial pattern of published peer‐reviewed research articles across Hindu Kush Himalaya

As shown by Figure 3, the research on HWC during the review period reveals an uneven pattern across the HKH. The largest number (87) of peer‐reviewed articles was published from India which accounts for only14% of the HKH area; the second largest number (85) came from Nepal (whose entire area is within the HKH), followed by Pakistan, Bhutan, and China. Very few studies were recorded from Myanmar (three) and Afghanistan (two) which take up 47% and 60%, respectively, of the HKH area. As for Bangladesh—with 9% of its area in the HKH—it recorded just one study. The districts with the largest number of publications from India were Pauri Garhwal and Chamoli in the state of Uttarakhand, while those from Nepal were Chitwan, Mustang, and Bardiya. In Pakistan, more articles came from the northern‐most district, while in the case of Bhutan, the largest number of research studies were from the Punakha district.

### 3.2 Spatial scale and theme

**FIGURE 4:**
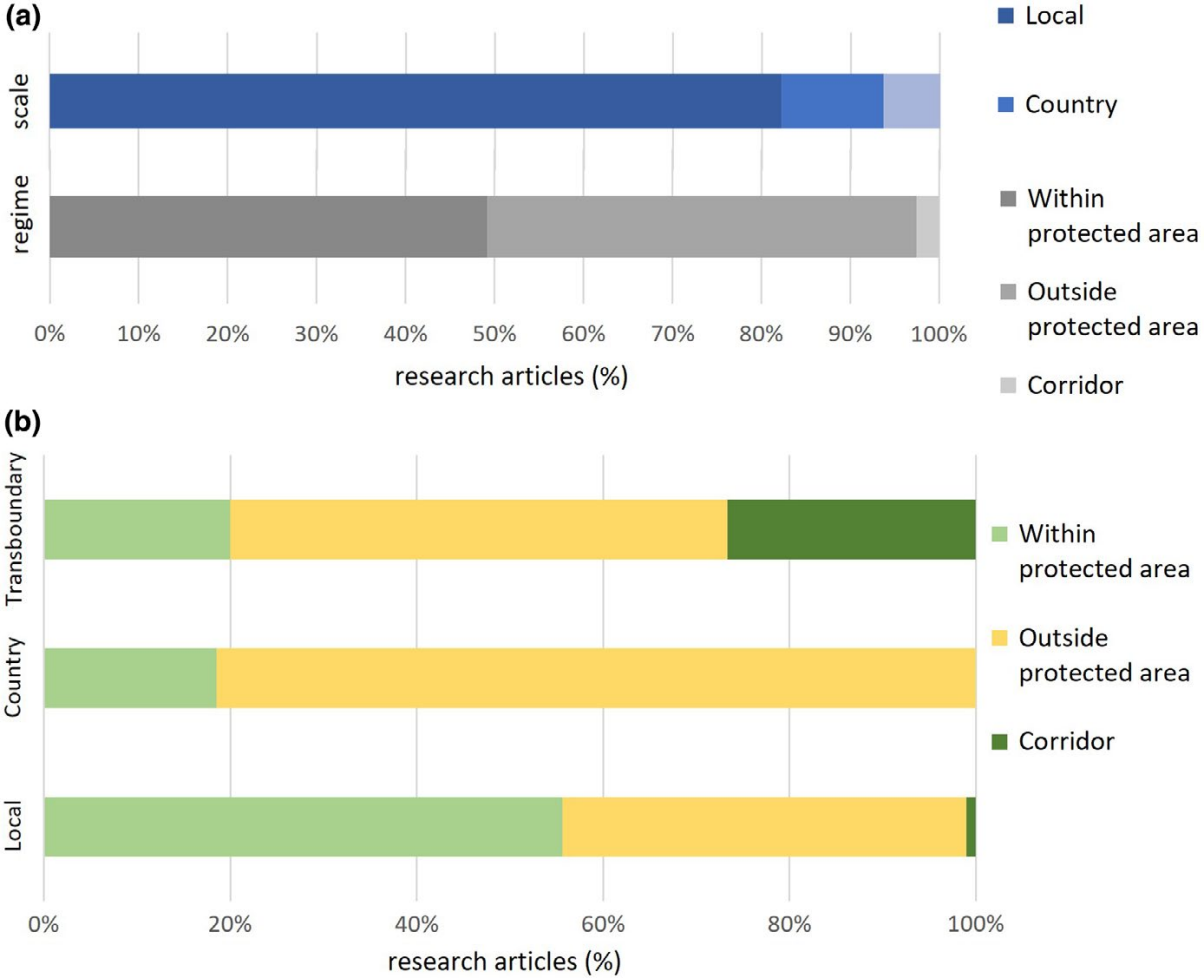
(a) Percentage of research articles according to scale (local, country, and transboundary) and regimes (within protected area, outside protected area, and corridors) of study sites and (b) Percentage of research articles conducted in various regimes (within protected area, outside protected area, and corridors) according to scale (local, country, and transboundary) of study sites

The research sites were also analyzed based on their scale—whether it was local, or was a country, or had a transboundary character (Martínez‐Harms & Balvanera, 2012)—and management regimes—whether it was a protected area (PA), wildlife corridor, or outside a PA. It was found that the majority (82%) of the research in the HKH on HWC were local‐level studies, followed by those at the country (12%) and transboundary levels (6%—Figure 4a).

About the management regimes of the study sites, nearly half (49%) of the studies were conducted outside PAs, such as in villages and towns; studies within and along PA boundaries, such as in and around wildlife sanctuaries, national parks, conservation areas, and biosphere reserves, covered 48%, while research within wildlife corridors accounted for 3% of the total publications.

A comparative analysis of the scale and regime of the study sites revealed that most local‐level studies (56%) were conducted within and along PA boundaries, followed by those (43%) outside PAs (Figure 4b). In the case of country‐level studies, most (81%) of them were conducted outside PAs. This is relevant since very few studies (19%) were conducted for protected areas within various parts of the country, qualifying as country‐level studies within PAs while none took place within wildlife corridors. About the transboundary‐level studies, they constituted the least number of publications, with most (53%) of the research taking place outside PAs, followed by 27% in the wildlife corridors and 20% within PAs.

**FIGURE 5:**
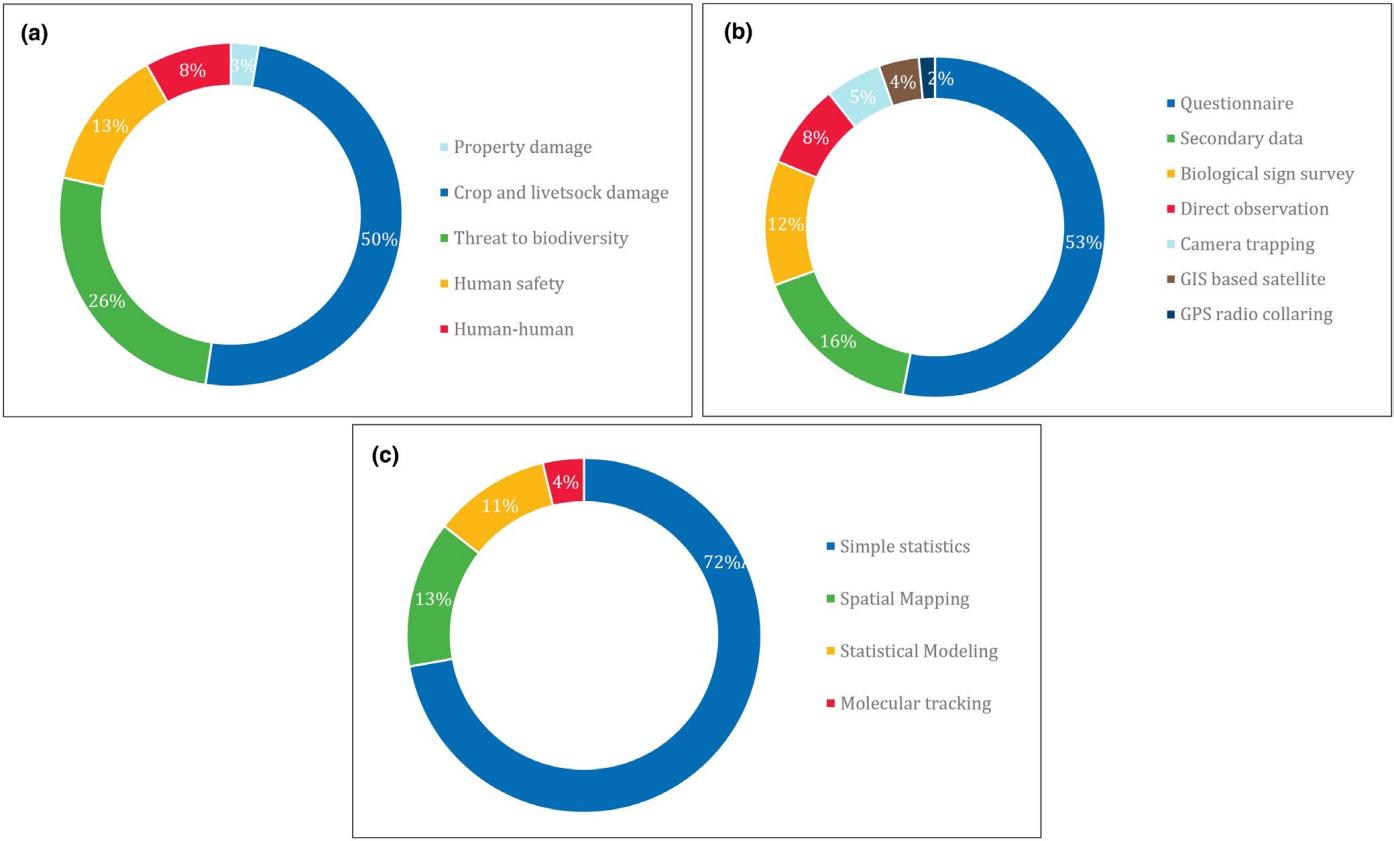
Percentage of research articles according to their (a) types of conflict, (b) methods used for collection of data, (c) methods used for analysis of data

In terms of the types of conflict, 50% of the articles discussed confrontations related to wildlife damaging crops and livestock; 26% focused on threats to biodiversity; 13% dwelt on aspects of human safety (by way of lethal attacks and the resultant psychological disruption); 8% examined human–human conflict arising mostly out of stakeholder disagreements (Figure 5a); and 3% of the pieces covered property damages involving destruction of built‐up structures and fences by the wild animals.

### 3.3 Research methods

Over half (53%) of the articles used data from interviews based on questionnaires and focus group discussions; about 16% used secondary data from reports, journal articles, and documents from government and nongovernment organizations; 12% carried survey results of biological samples like hair, scat, scrapes, and footprints of wild animals; 8% relied on direct observations or sightings of animals; 5% depended on camera trapping; 4% on GIS‐based satellite data such as for climate, elevation, and land‐cover maps; and 2% used the GPS radio‐collaring method for the data collection on HWC studies in the region (Figure 5b).

The various approaches adopted in these studies to analyze data included the use of simple statistics that involved the calculation of percentage, mean, and standard deviation, as well as the use of *t* test—this approach was illustrated in 72% of the research articles; 13% depended on spatial mapping using GIS tools; 11% on statistical modeling techniques like logistic regression and generalized linear mixed models; and 4% relied on DNA‐based molecular tracking of biological samples to understand the dietary habits of the relevant wild animals (Figure 5c).

### 3.4 Focal species or taxonomical group

The classification of studies based on wildlife taxonomical groups (Peterson et al., 2010) revealed that 46% of the research focused on large carnivores (Table 3) such as the snow leopard, the common leopard, the Royal Bengal tiger, the gray wolf (*Canis lupus*), and the dhole (*Cuon alpinus*); 27% dwelt on omnivores such as the Asian bear, the brown bear (*Ursus arctos*), the monkey (*Macaca mulatta*), and the boar (*Sus scrofa)*; 16% concentrated on mega herbivores such as the elephant and the one‐horned rhinoceros (*Rhinoceros unicornis*); 7% studied crop raids and illegal poaching of herbivores such as ungulates and antelopes (7%); and 4% focused on meso‐mammals like the porcupine (*Hystrix brachyuran)* and the marmot (*Marmota himalayana*). We also found 1% mention of medium carnivores like the Himalayan lynx (*Lynx isabellinus*) and the leopard cat (*Prionailurus bengalensis*), and 0.4% references to small carnivores, particularly the yellow‐throated marten (*Martes flavigula*).

**TABLE 3:** Percentage of research articles relating to various wildlife taxonomic groups

Categories	Percentage of research articles
Large carnivores	45.9
Omnivores	26.7
Mega herbivores	16.1
Herbivores	6.7
Meso mammals	3.5
Small carnivores	1.2

### 3.5 Drivers for the conflict

**FIGURE 6:**
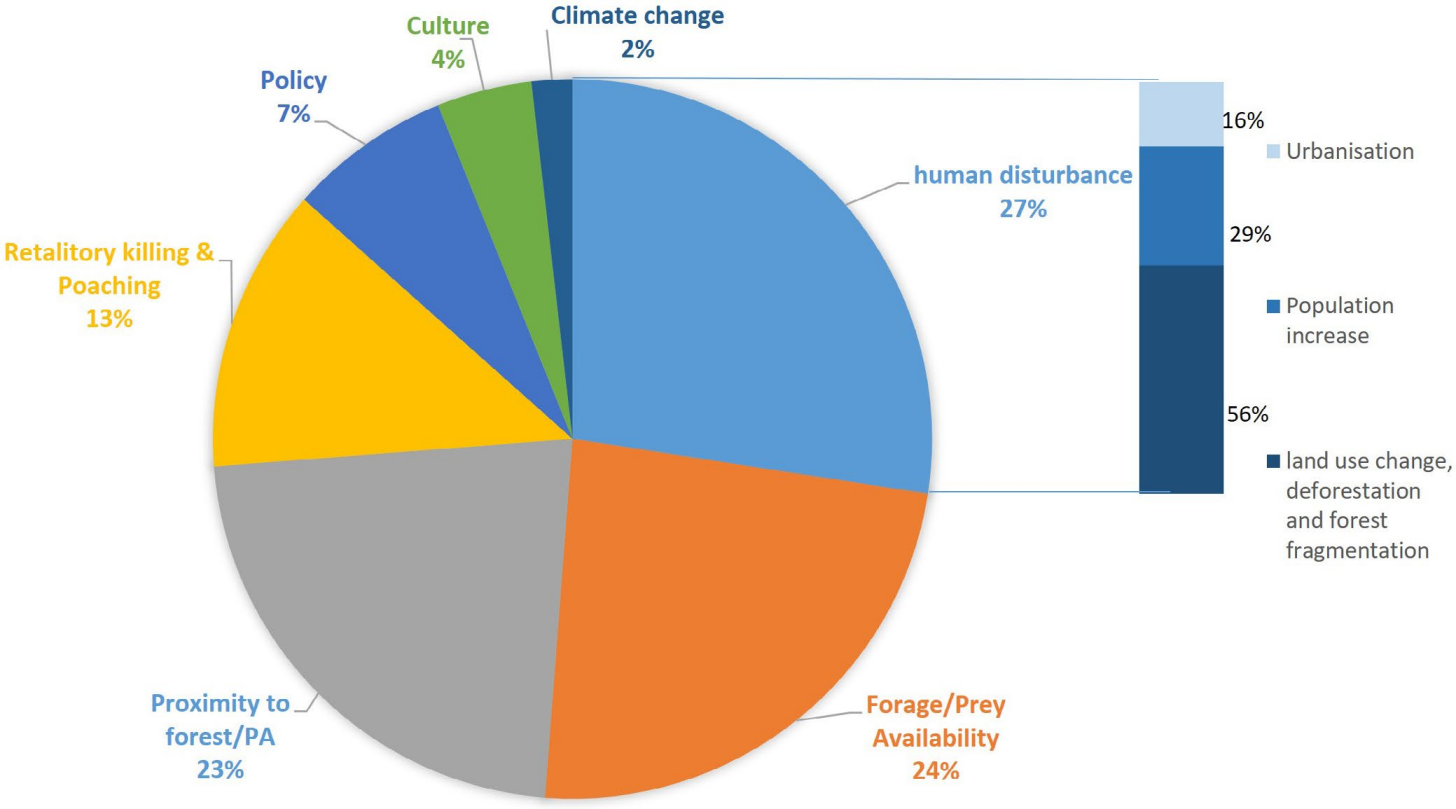
Drivers of human–wildlife conflict related change considered by research articles expressed in percentages

Over half (60%) of the articles considered at least one driver of change triggering HWC (Figure 6). The most frequent (27%) cause of disruption reported was disturbance of the natural landscape due to human population growth, rapid urbanization, and widespread land‐use changes; 24% of these studies centered on shortage of food such as forage and wild preys; 23% discussed the proximity of human settlements to PAs, which enabled the forest communities to access them for firewood and herbal medicines, thereby leading to conflicts between these communities and the wildlife around them; 13% of the research articles were on retaliatory killing and illegal poaching of wild animals; 7% were on changes in conservation policies; 4% dealt with culture and its shifting patterns, while only 2% of the articles deliberated on how climate change was an important driver of HWC in the region.

### 3.6 Management interventions

**FIGURE 7:**
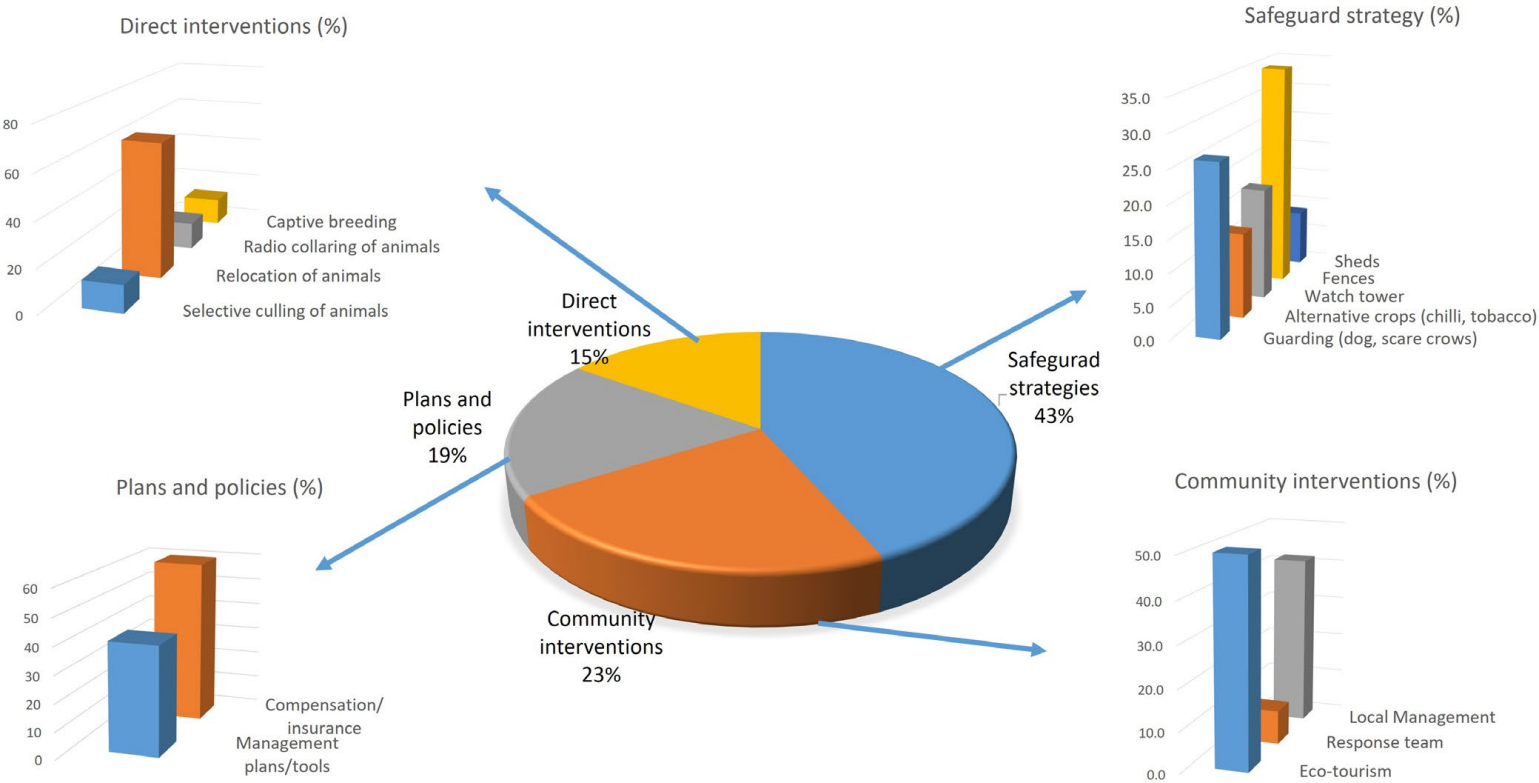
Management actions on human–wildlife conflict recommended by research articles expressed in percentages

As for HWC mitigation strategies, only 20% of the selected literature specifically discussed or recommended them (Figure 7); and each of these articles recommended two or more management actions or interventions. The most commonly recommended interventions (43%) were protecting fields and livestock by deploying watchdogs and scarecrows, and constructing sound sheds or corrals for the livestock; growing alternative cash crops like tea, chilli, and tobacco; and building electric or bio fences, and water towers. Among other suggestions, 23% of the articles recommended community interventions in the form of promoting ecotourism and setting up response teams. Some (19%) articles put forth management plans to tackle HWC and also proposed improvements in compensation policies. A few (15%) others recommended interventions such as relocation, selective culling, radio‐collaring, and captive breeding of wild animals (Figure 7).

### 3.7 Inclusion of perception, attitude, and gender aspects

About 22% of the articles investigated people's perception and attitude toward HWC, while only 5% dwelt on the issue of gender; and out of this 5%, only one article focused exclusively on the role of gender in HWC while the rest considered it as one among the other factors influencing these conflicts.

### 3.8 Co‐occurrence of keywords; coauthorship linkages; and country collaborations

**FIGURE 8:**
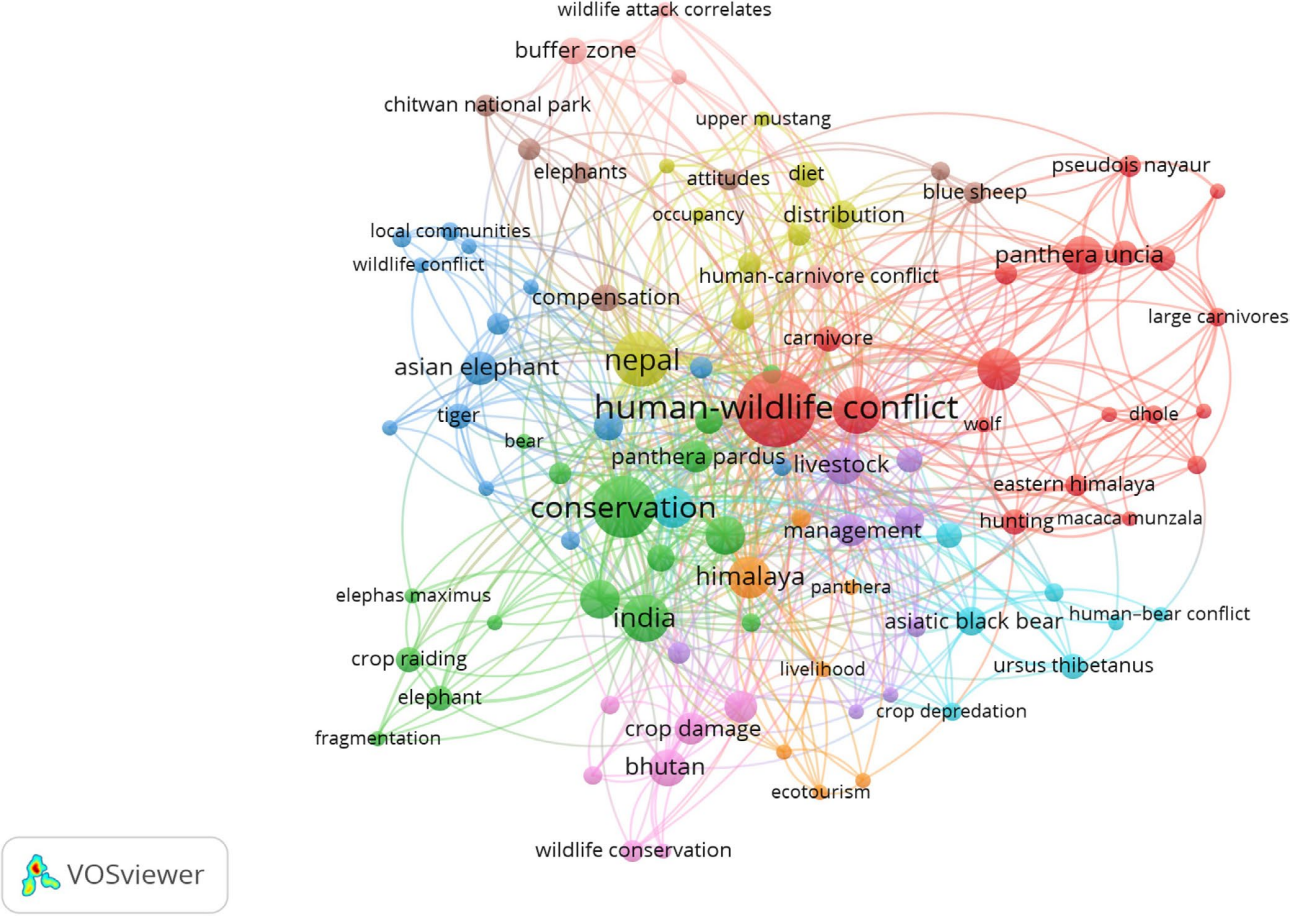
Network of Keywords co‐occurrence for human–wildlife conflict research in the Hindu Kush Himalaya

A total of 681 keywords were found in the selected HWC literature, out of which 533 appeared only once. Some of them were “activity pattern”, “anthropogenic threats”, “agro‐pastoralism”, “aggressive behavior”, and “alternatives” (Figure 8). The keyword that occurred most frequently (48 times) was, expectedly, “human–wildlife conflict”, followed by “conservation” (32 times) and “Nepal” (25 times). The total strength of the co‐occurrence link or the total link strength of these keywords was high compared with the keywords with low occurrence. “India”, “livestock depredation”, “snow leopard”, “Himalaya”, and “Asian elephant” were among the top 100 keywords with the highest total link strength apart from the keywords with high occurrence.

**FIGURE 9:**
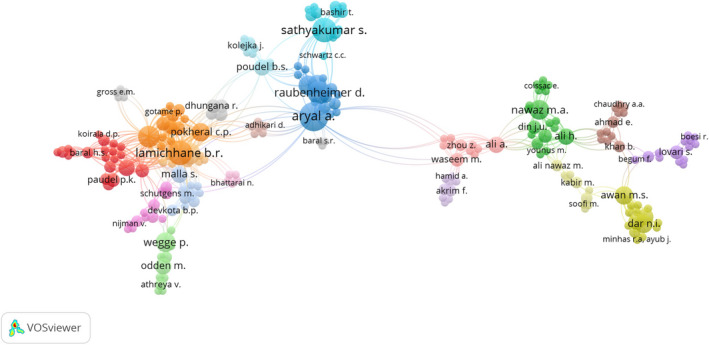
Network of coauthorship between researchers on human–wildlife conflict in the Hindu Kush Himalaya

As many as 640 individual authors contributed to the research on HWC in the HKH during the study period. Among these, only 228 authors were interconnected, to each other forming 21 clusters of authors (Figure 9). As for coauthorship, 22% (*n* = 52) of the papers were written by two authors, while 4% (*n* = 9) of them involved 10 authors. And, about 10% (*n* = 25) of the articles were written by a single author. The dataset also contains an article by 14 authors, the highest number, and one by 12 authors. Further, the study looked into the most sizeable contribution made by authors to research on HWC in the HKH; it was found that four authors—A. Aryal, B.R. Lamichhane, C. Mishra, and S. Sathyakumar—were most prominent constituting 2% of the total research on the subject in the HKH. The other sizeable contributions came from researchers D. Raubenheimer, M. Dhakal, and N. Subedi. The list of the top 15 authors in terms of the total number of articles authored and coauthored is presented in Table 4.

**TABLE 4:** Top 15 authors in HKH‐HWC research

Author name	Number of articles	Primary affiliation^a^	Number of collaborating authors	First author article(s)	Citations
A. Aryal	13	University of Sydney, Australia	60	8	137
B.R. Lamichhane	9	National Trust for Nature Conservation, Nepal	67	4	19
C. Mishra	9	Nature Conservation Foundation, India	22	2	444
S. Sathyakumar	9	Wildlife Institute of India, India	28	0	52
D. Raubenheimer	8	Massey University, New Zealand	37	0	125
M. Dhakal	7	Ministry of Forests and Environment, Nepal	45	0	23
N. Subedi	7	National Trust for Nature Conservation, Nepal	63	0	15
Y.V. Bhatnagar	6	Nature Conservation Foundation, India	20	0	224
D. Brunton	6	Massey University, New Zealand	26	0	117
R.K. Maikhuri	6	G.B. Pant Institute of Himalayan Environment and Development, India	24	3	283
S. Nautiyal	6	Centre for Spatial Science, Japan	24	0	283
M.A. Nawaz	6	Quaid‐i‐Azam University, Pakistan	21	1	61
K.S. Rao	6	University of Delhi, India	24	3	283
K.G. Saxena	6	Jawaharlal Nehru University, India	24	0	283
P. Wegge	6	Norwegian University of Life Sciences, Norway	13	1	116

^a^ The affiliations of the authors are based on their latest article available in the database.

**FIGURE 10:**
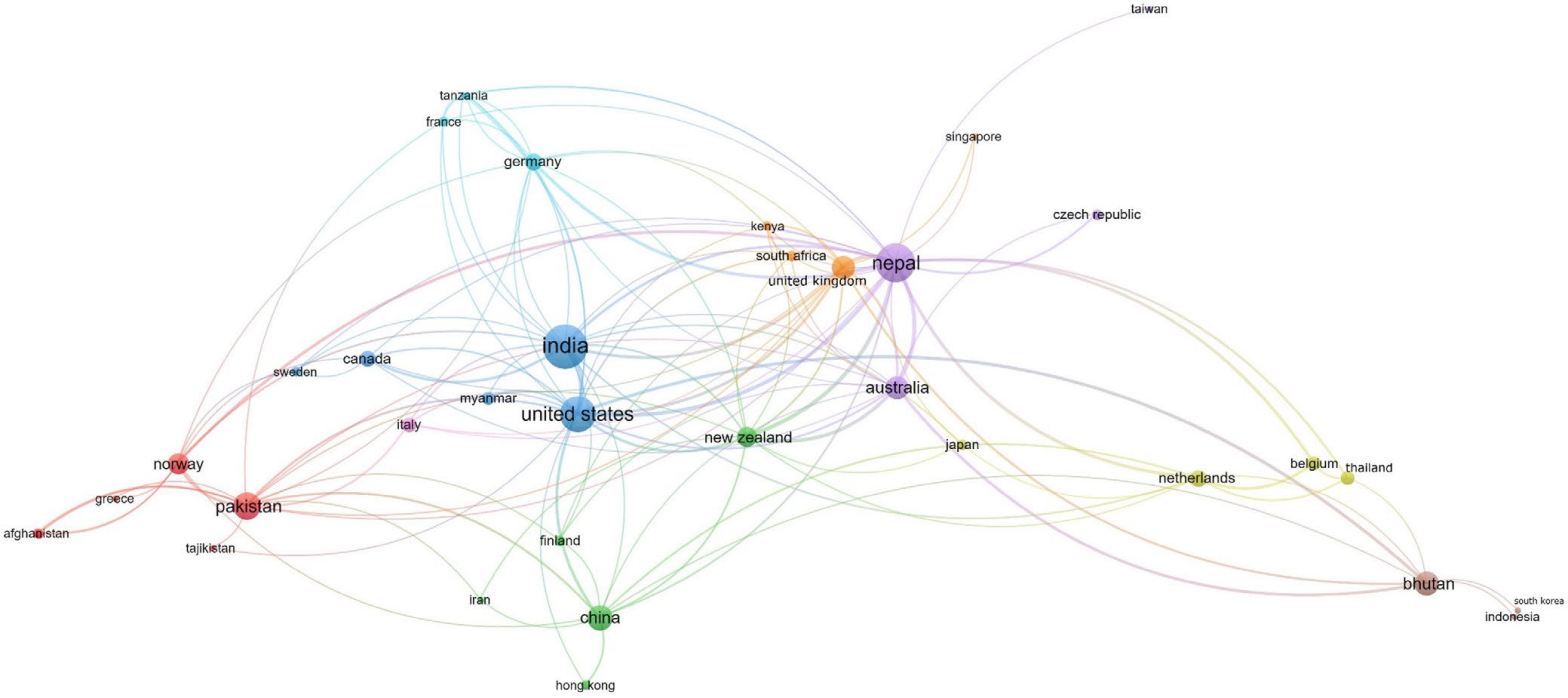
Network of country collaboration on human–wildlife conflict research in the Hindu Kush Himalaya

The research on HWC in the HKH has had some degree of country partnership networks (Figure 10). Our study identified authors from 36 regions—17 from Asia, 11 from Europe, and the remaining from Africa, North America, Australia, and Oceania—being involved in such collaborative exercises. Notably, there was no collaboration with countries in South America. In terms of the number of articles published by the collaborating countries, India stood at the top (*n* = 87), followed by Nepal (*n* = 64) and the United States (*n* = 53). The size of this circle is indicative of the number of articles published by each country, as illustrated in VOSviewer. Among the nine clusters of countries in the collaboration network, Nepal had the highest number of collaborations (also known as the total link strength; in this case, 81 links) with other countries, followed by the United States and India. The top authors from Nepal were affiliated with institutions in the developed countries, while the ones from India were mainly related to government organizations. Nepal collaborated with three other HKH countries—India, Pakistan, and China—as well as with other countries from Asia, America, Africa, and Europe. The United States, with the second‐highest collaboration links (*n* = 65), partnered with authors from five HKH countries—Bhutan, China, India, Nepal, and Pakistan—and with other international collaborators. The third on the list was India, which entered into collaborations with three HKH countries—China, Myanmar, and Nepal—and was also involved in a few other international partnerships. Interestingly, one study from the HKH region of Bangladesh was found to be not part of the co‐authors’ country collaboration network.

## 4 DISCUSSIONS

### 4.1 Temporal and spatial pattern

The growing interest of scholars in wildlife conflict and management (Seoraj‐Pillai & Pillay, 2017) is reflected in the global increase in the number of research articles since the 1980s on the science of applied ecology related to conservation and biodiversity (Anderson et al., 2021). Though the research publications on HWC from the region remained low in the initial phases, their rates climbed considerably by 57% in the last decade. This trend coincided with an increase in the severity and frequency of HWC in several parts of the HKH (Inskip & Zimmermann, 2009) as a result of growing human dependency on natural resources and the degradation of wildlife habitats (Manral et al., 2016; Xu et al., 2019). About 465 human fatalities were reported from Nepal between 2010 and 2014, which highlighted the gravity of the problem (Acharya et al., 2016). Meanwhile, there was a considerable increase in the population of livestock, especially goats, in the mountains of Bhutan, India, and Pakistan, which made them vulnerable to attacks (Tulachan, 2001). On the contrary, Nepal also made significant progress in terms of conservation—for instance, by reversing the decreasing trend—which lasted for three consecutive years—in the population of rhinoceros; this came about by achieving the target of zero poaching (Acharya, 2016).

As indicated earlier, most of the research on HWC in the HKH emerged from India and Nepal, which constituted 72% of the total publications. Though only 14% of India's area lies within the HKH, the number of publications from that country was the highest among all. At the other extreme were Myanmar and Afghanistan, which, despite having half of their territory within the HKH, accounted for only a limited number of studies on HWC. In the case of India, its districts of Pauri Garhwal and Chamoli in Uttarakhand drew major attention from the researchers since these districts were known to be among those with the highest number of HWCs, especially involving large carnivores (Agarwal et al., 2016; Gupta and Bhatt, 2009; Naha et al., 2018; Sondhi et al., 2016). Another focal point of interest for the researchers was Nepal's Chitwan district where several studies were conducted in and around Chitwan National Park (Lamichhane, 2019; Sapkota et al., 2014). Here, it was be noted that almost half of the HWC studies which came under this review took place within and along PAs. In the HKH, there are 545 PAs with varying degrees of protection and status, and these occupy about 40% of the region's terrestrial land (Chettri et al., 2008; Chaudhary et al.—yet to be published). However, these PAs, home to many significant animal species, have been under tremendous pressure from livelihood‐dependent communities (Gu et al., 2020; Sharma & Yonzon, 2005). The intensification of land‐use for agriculture and the rearing of livestock within and along the periphery of PAs have increased instances of crops and livestock being attacked by wild animals. As for studies on HWC in the wildlife corridors, these were few and far between—only about 3% of the overall research during the review period. The corridors mentioned in these studies were the Rajaji‐Corbett Corridor, the Laljhadi‐Mohana Corridor, the Khata Corridor, and the Wakhan Corridor.

### 4.2 Spatial scale and theme

As indicated earlier, since most of the studies on HWC in the region were local, they were not able to take up the issue on a transboundary level. While it is a fact that instances of HWC in and around PAs and corridors in a transboundary complex such as the Terai Arc Landscape (TAL) between India and Nepal have been reported (Balodi & Anwar, 2018; Jasmine et al., 2015), no significant intercountry collaboration has yet taken place to tackle the problem (MoFSC, 2015). This lack of cross‐border partnerships and understanding about the transboundary nature of HWC is a glaring gap in HWC research in the region; not enough studies have explored the migratory bent or the compulsion of wild animals which takes them across national borders (ICIMOD, WCD, GBPNIHESD, RECAST 2017; Sharma et al., 2020).

This review establishes that half of the HWC research conducted in the region has focused on the damage caused to crops and livestock by wild animals. In this regard, it was found that Bhutanese households incurred an annual loss in income by around 25% due to crop raids by foraging animals (Tobgay et al., 2019) and by about 10%–19% because of livestock depredation (Jamtsho & Katel, 2019). Such huge losses pose a challenge to any country's local food system, while also adversely affecting the livelihood of its people and their food and nutrition security (Sharma et al., 2020). Hence, to address this problem, a large volume of research concentrated on understanding the foraging characteristics of animals and the pattern of livestock depredation; the studies also assessed habitats (both animals in conflict and their preys) and discussed ways of how humans and wildlife could live peacefully with each other (Aryal et al., 2015; Bargali & Ahmed, 2018; Bhattacharjee & Parthasarathy, 2013; Rao et al., 2002). Besides stressing on conserving endangered species, the researchers emphasized the threat to biodiversity in the region owing to illegal hunting, killing, and trade in animal body parts (Bhattarai & Kindlmann, 2012; Rao et al., 2010; Rimal et al., 2018; Thapa, 2014; Uprety et al., 2021). Globally, the researchers highlighted the need for shifting attention toward human–wildlife coexistence, a sustainable state wherein humans and wildlife coadapt to live in shared landscapes (König et al., 2020; Peterson et al., 2010). Although the disharmony between biodiversity conservation efforts and communities affected by conflict is vast, smaller amount of research (15% of the total) has taken place in this area. Discord involving disagreements among communities, stakeholders, and policymakers is an area that requires an understanding of the socio‐political processes that affect conservation management (Rastogi et al., 2014). These disagreements between the region's indigenous forest‐dependent communities—who feel a sense of stewardship over the forests and grasslands—and the forest departments over governmental policies on resource utilization and compensation impede effective management and resolution of issues related to HWC.

### 4.3 Research methods

The researchers collected about 53% of primary‐level data from the region through household surveys and focus group discussions, and also supplemented their research with the existing secondary data. Only a small percentage of the data was collected with the aid of GPS radio‐collaring, GIS‐based satellite images, and camera trapping. Though the data on HWC in the region are not generally deficit, it is mostly skewed toward understanding of the human dimension of HWC. There is a gap in the use of better technologies for data collection vis‐à‐vis the analysis of the patterns of interaction of wildlife with their surroundings; the same is mostly true in the case of studying their migratory routes and dietary habits. Mostly, in about 73% of the cases, the data analysis was based on simple statistics; while the rest depended on spatial mapping and modeling. The use of advanced methods such as DNA‐based molecular tracking of biological samples to understand the dietary habits of wild animals contributed only a small portion to HWC research in the region. It is important here to state that inferences on the feeding behavior of wild animals help in understanding how their food habits influence the ecosystem; these inferences also give insights into the wild animals’ relationship with the local livestock, thereby aiding in the establishment of reliable management programs that can pre‐empt instances of HWC.

### 4.4 Focal species or taxonomical groups

As mentioned earlier, during the review it was found that the majority (46%) of the research on the conflict between humans and wildlife in the HKH dealt with large carnivores, with the snow leopard being the one that was researched the most (20%), followed by the leopard (18%), and the tiger (15%). The relatively large number of research articles on the conflict between snow leopards (commonly found in the high mountains of China and South Asia) and humans reflects the spate of such incidents in the Himalayas and the Karakoram range since 1994 (Rashid et al., 2020); this had to do with the dwindling number of the snow leopard's wild preys which forced it to indulge in retaliatory attacks against the high‐mountain communities and pastoralists (Chetri et al., 2019; Rosen et al., 2012). As for studies on human conflicts with other carnivores such as wolves and dholes—predators inhabiting the central‐western parts of the Himalayas and the eastern Himalayas, respectively (Johnsingh et al., 2007; Xu et al., 2015)—they were much less compared with those involving snow leopards. Another conflict that attracted significant research attention was the one involving omnivores (bears, monkeys, and boars) and mega herbivores (elephants and rhinoceros), and particularly their propensity to attack livestock and crops. In 2008, researcher P. Yonzon pointed out that in Nepal alone, up to 20,000 people in the southern lowlands were caught in conflicts with elephants, thereby suggesting that confrontations with mega herbivores were an issue of huge concern. However, while the issue of conflict with large carnivores, omnivores, and mega herbivores drew much research attention, the same could not be said of conflicts involving small carnivores, meso‐mammals, birds, and reptiles—only about 1% and 0.4% of the studies covered carnivores of medium and small sizes, respectively. This was because they were perceived to pose less danger than large carnivores, though Sunar et al. (2012) found out that the yellow‐throated marten alone was responsible for half of the attacks on village livestock in and around Senchal Wildlife Sanctuary in Darjeeling, India. This would have to do with the fact that these small carnivores live within a narrow habitat range (commonly within 1,700–2,000 masl) and their survival in many parts of the HKH is threatened by degradation of habitat, shortage of food in the wild, and poaching. Similarly, studies on HWC concerning meso‐mammals, birds, and reptiles have been rather sparse in the region even though porcupines, peafowls, marmots, and civets are known to be a menace to many farmlands in the HKH (ICIMOD, WCD, GBPNIHESD, RECAST 2017; Pradhan, 2018).

### 4.5 Drivers of conflict

Since the HKH is one of the most affected areas in terms of human and animal deaths due to HWC (Torres et al., 2018), it is important to understand the dynamics that drive the relationship between humans and wildlife. Most often, the factors are area‐specific and highly complex; they are known to hinge on the socioecological behavior of humans, the nature of wildlife, and the availability of resources (Dickman, 2010; Nyhus, 2016). Most articles point to habitat disturbance—as a result of land‐cover change and forest fragmentation, as well as due to population growth (leading to pressure on natural resources), urbanization, and industrialization—as a major cause behind HWC (Reshamwala et al., 2018). Some studies say that the unavailability of fodder in the wild (Acharya et al., 2016) and the presence of human habitation in the vicinity of most forests have pushed the wild animals into a corner from where it becomes inevitable that they resort to attacks on humans, their livestock, and their crops.

As for research literature discussing climate change as a factor influencing HWC in the region, only about 2% of the studies took up the issue, even though that the HKH is a hotspot in terms of climate change (Sharma et al., 2019). Among those few studies, the one by Bashir et al. (2018) states that climate change affects the phenology of forage in the wild and causes a shift in habitat whereby the animals come into conflict with the nearby communities.

### 4.6 Management interventions

Some of the literature describes various traditional management techniques of mitigation that have been in practice in the region for decades. The communities in the HKH rely on watchdogs, guards, and fences to safeguard their livestock and crops. In Nepal, farmers find guarding from watchtowers with flaming sticks and noise effective in scaring away elephants, while barriers like net wires and trenches are useful against smaller mammals (Dhakal & Thapa, 2019). As suggested by the SAARC Forestry Centre in 2014, many local communities have installed electric and solar fences to ward away predatory wildlife. However, these actions alone do not prevent attacks on crops and livestock; the farmers need to be on a constant vigil and the barriers need to be well maintained and repaired when required. Interestingly, a recent study (Perrotton et al., 2017) notes no significant difference between guarded and unguarded fields in terms of revenue loss from crop raids. But what usually works is a community‐based guarding system, like in the case of large farming blocks, as is in place in parts of the Indo‐Gangetic plains (Gross et al., 2019). A few articles state that community intervention and adaptation methods, such as by way of ecotourism and local management of resources, have the potential to uplift local livelihoods as well as sustainably develop ecosystem services (Bhalla et al., 2016).

Some of the researchers have also laid stress on formulating proper compensation policies and programs, which, they say, discourage retaliatory killings and build community support for conservation (Agarwala et al., 2010; Naughton‐Treves et al., 2003; Persson et al., 2015). However, these compensation schemes are often vulnerable to corruption and long administrative delays; they also fail to account for transaction costs; further, in many HKH countries, the compensation policies are rather restricted in scope, as they are targeted only toward losses from large carnivores and mega herbivores (Upadhyay, 2013). In a similar vein, the conflict–response system of various government agencies in the HKH could be strengthened through a better mechanism for complaint submission by conflict victims, the lowering of transaction costs, the inclusion of relevant conflict‐prone species in the scheme of things, and the standardization of policies (Karanth et al., 2018).

### 4.7 Inclusion of perceptions, attitude, and gender

Several research articles also studied the HKH people's perception and attitude toward conflict with wildlife and how to manage it. Such a study of the perception and attitude of the local communities is important to ensure that wildlife management policies are effective and also sensitive to local conditions. Though most studies revealed that the communities nursed a negative attitude toward conservation authorities, such as officials of national parks, they did express a positive attitude toward conservation and coexistence, guided by religious and cultural beliefs (Anand et al., 2018; Xu et al., 2015). Meanwhile, on the issue of gender, while it has been reported that risks and priorities in terms of HWC are seen differently by women and men (Gore & Kahler, 2012), this is a deficient area of research in the HKH. Another area of research that has to be explored substantially relates to the study of how humans and wildlife can coexist peacefully if effective wildlife management practices are in place and a mechanism developed for human–wildlife interface through appropriate tools and techniques.

### 4.8 Co‐occurrence of keywords, coauthorship linkages, and country collaboration

In this literature review, when we analyzed keywords, HWC featured prominently along with associated species, type of damage including hotspot areas. In the authorship, we noted, as mentioned earlier, that out of the 640 authors who worked on HWC, only 228 of them coauthored HWC publications in the HKH, thereby forming a collaborative network (Figure 9). We also noted that the authors in this network (Figure 10) were less interconnected when compared to other areas of research in the HKH, such as in the field of ecosystem services (Kandel et al., 2020). Further, we found out that there were no networks of HWC studies among the HKH countries of India, Pakistan, Myanmar, and Bangladesh. Since HWC has an intrinsic transboundary character, it calls for regional cooperation at multiple levels—be it in research or for administrative purposes—such as in the form of trans‐frontier complexes like TAL that covers areas in India and Nepal that have been highly affected by human–wildlife confrontations. In this regard, a recent study by Sharma et al. (2020) is the first instance of transboundary research collaboration involving authors from Bhutan, India, and Nepal in the landscape of Kangchenjunga. Such regional‐level HWC studies would also be useful for transboundary areas such as Karakoram, Pamir Knot, and the Kailash Sacred Landscape in the HKH (Din et al., 2019; Hussain et al., 2018).

## 5 CONCLUSION

This study evaluated the status, analyzed trends, and identified gaps in HWC research in the HKH. It is evident that the HKH is one of the hotspots of HWC, having suffered severe losses in terms of both human and animal lives, as well as by way of crops and livestock, but there is yet no silver‐bullet option available to resolve the issue. Since the literature on HWC has been rather disproportionately focused on geographical and thematic topics such as PAs, large carnivores, and mega herbivores, a huge knowledge gap exists in this field of study. This warrants more and meticulous analyses of several aspects of HWC, especially in the western and far‐eastern Himalayas, and these should mainly deal with mitigation options and patterns of human–wildlife interaction. To date, most studies have revolved around localized PAs. But the escalating cases of HWC in the HKH demand greater emphasis on studies of a larger scale at the transboundary level, whereby wildlife corridors also come into the picture. This is also a time to reinforce the methodologies and precision of the studies in the HKH through the adoption of advanced technological tools such as camera traps and DNA‐based molecular trackers.

Most studies on HWC in the region have been on large mammals; however, given the fact that small mammals and birds also inflict damages on crops and livestock, their roles ought to be investigated in depth in future studies. Another area of study that requires close attention relates to the connection between climate change and HWC, especially because the HKH is prone to habitat degradation and shift in species habitat. Equally important is the aspect of gender which has not yet been adequately captured in the research on HWC in the region. Finally, and most importantly, it is the transboundary nature of HWC in a region that has a common ecosystem; hence, there is an urgent need for better research collaboration among the HKH countries that would also enable the academically weak countries to be on a stronger footing in tackling HWC.

In summing up this review of research literature on the conflicts between humans and wildlife in the HKH, it has to be stated that while studies in this sphere have gathered pace, there are yet vital areas that need to be explored further—only then can we be better prepared to mitigate this menace.

## CONFLICT OF INTEREST

The authors declare no conflict of interest.

## AUTHOR CONTRIBUTIONS

**Prashanti Sharma:** Conceptualization (supporting); Data curation (lead); Formal analysis (lead); Methodology (lead); Writing‐original draft (lead); Writing‐review & editing (equal). **Nakul Chettri:** Conceptualization (lead); Data curation (supporting); Formal analysis (supporting); Investigation (lead); Methodology (supporting); Resources (lead); Writing‐original draft (supporting); Writing‐review & editing (supporting). **Kesang Wangchuk :** Conceptualization (supporting); Data curation (equal); Formal analysis (supporting); Investigation (lead); Methodology (equal); Writing‐original draft (supporting); Writing‐review & editing (supporting).

## Data Availability

The datasets generated as bibliography during the current study are available from the corresponding author on reasonable request.
